# Acid-sensing ion channels are expressed in the ventrolateral medulla and contribute to central chemoreception

**DOI:** 10.1038/srep38777

**Published:** 2016-12-09

**Authors:** Nana Song, Ruijuan Guan, Qian Jiang, Comron J. Hassanzadeh, Yuyang Chu, Xiaomei Zhao, Xia Wang, Dawei Yang, Qijun Du, Xiang-Ping Chu, Linlin Shen

**Affiliations:** 1 Department of Physiology and Pathophysiology, School of Basic Medical Sciences, Fudan University, Shanghai 200032, China; 2 Division of Nephrology, Zhongshan Hospital, Fudan University, Shanghai 200032, China; 3 Department of Basic Medical Science, School of Medicine, University of Missouri – Kansas City, Missouri 64108, USA; 4 Division of Pulmonary Medicine, Zhongshan Hospital, Fudan University, Shanghai 200032, China; 5 School of Mental Health, Qiqihar Medical University, Qiqihar, Heilongjiang 161006, China; 6 Shanghai Key Laboratory of Medical Imaging Computing and Computer Assisted Intervention, Fudan University, Shanghai 200032, China

## Abstract

The role of acid-sensing ion channels (ASICs) in the ventrolateral medulla (VLM) remains uncertain. Here, we found that ASIC1a and ASIC2 are widely expressed in rat medulla, and the expression level is higher at neonatal stage as compared to adult stage. The two ASIC subunits co-localized in medualla neurons. Furthermore, pH reduction triggered typical ASIC-type currents in the medulla, including the VLM. These currents showed a pH_50_ value of 6.6 and were blocked by amiloride. Based on their sensitivity to psalmotoxin 1 (PcTx1) and zinc, homomeric ASIC1a and heteromeric ASIC1a/2 channels were likely responsible for acid-mediated currents in the mouse medulla. ASIC currents triggered by pH 5 disappeared in the VLM neurons from ASIC1^−/−^, but not ASIC2^−/−^ mice. Activation of ASICs in the medulla also triggered neuronal excitation. Moreover, microinjection of artificial cerebrospinal fluid at a pH of 6.5 into the VLM increased integrated phrenic nerve discharge, inspiratory time and respiratory drive in rats. Both amiloride and PcTx1 inhibited the acid-induced stimulating effect on respiration. Collectively, our data suggest that ASICs are highly expressed in the medulla including the VLM, and activation of ASICs in the VLM contributes to central chemoreception.

Central chemoreception plays an important role in the regulation of respiration and maintaining acid-base balance within the internal environment^1^. Protons are the effective stimulus for chemoreception, however, the ion mechanisms of central chemoreception are poorly understood. Though there are many candidates of pH-sensitive channels involved in the chemoreception, their effects on regulation of breathing remain uncertain^2^. Recently, protons have been identified as neurotransmitters in the brain^3^ and acid-sensing ion channels (ASICs) act as key receptors for extracellular proton sensing in both central and peripheral neurons^4^,^5^,^6^. However, little is known about the role of ASICs in central chemoreception.

ASICs are encoded by four genes for at least six transcripts including ASIC1a^7^, ASIC1b^8^, ASIC2a^9^, ASIC2b^10^, ASIC3^11^, and ASIC4^12^,^13^. Homomeric or heteromeric trimers form these functional ion channels which respond to proton gradients with different sensitivities^14^,^15^. ASIC2b and ASIC4 cannot form functional homomeric channels^10^,^12^,^13^, but they can associate with other ASIC subunit(s) to form functional heteromeric ASICs^15^,^16^,^17^. Among all ASIC subunits, ASIC1a and ASIC2 are the two most abundant subunits within the central nervous system (CNS) and play important roles in physiological and pathophysiological conditions^18^,^19^. ASIC1a is widely expressed in the CNS including the cortex, striatum, hippocampus, amygdala and hypothalamus^3^,^20^,^21^. They play an important role in learning/memory^22^,^23^, fear-related behaviors^24^, pain^25^, depression^26^, seizure termination^27^ and contribute to neuronal damage after cerebral ischemia^28^,^29^,^30^. ASIC2 channels have also been detected in the CNS^31^ and are involved in the integrity of cardiovascular function^32^,^33^. Expression of ASIC3 in the CNS was reported in the hypothalamus^34^, but its level is much lower compared to ASIC1a and ASIC2^35^,^36^. Its function in the brain however, remains undetermined.

Previous studies have shown that ASICs in the lateral hypothalamus and the nucleus of the solitary tract (NTS) contribute to central regulation of respiration^37^,^38^. The ventrolateral medulla (VLM) is a well-known region containing chemoreceptors. However, little is known about the expression and functional role of ASICs in the VLM. Thus, we hypothesize that ASICs are expressed in the VLM, and they contribute to central chemoreception.

To test this hypothesis, we studied ASIC expression in the rat medulla and acid-activated currents in the mouse medulla. To address the functional effect of ASIC activation, we performed i*n vivo* microinjection of acidic artificial cerebrospinal fluid (ACSF) in rats. Our data revealed an important role of ASICs in regulating central chemoreception in the medulla.

## Results

### Expression of ASIC1a and ASIC2a in neonatal and adult rats

**Figure 1:**
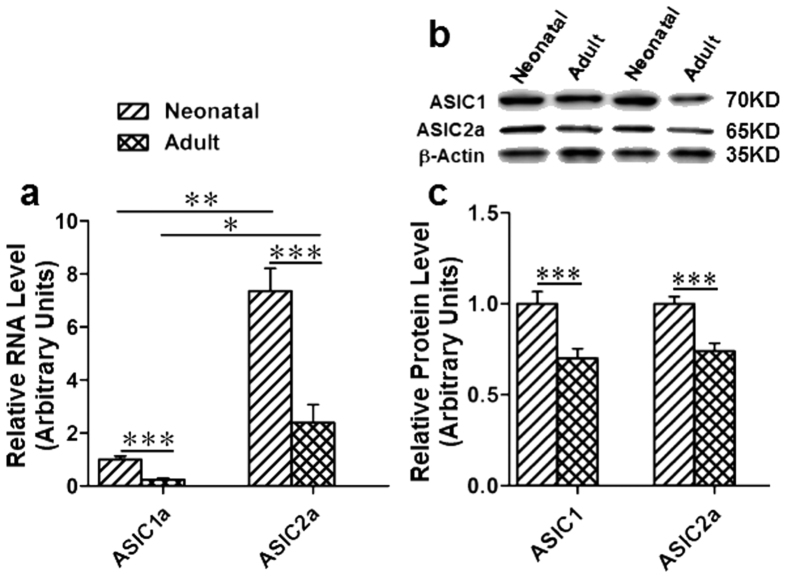
Expression of ASIC1a and ASIC2a in the medulla of adult and neonatal SD rat. **(a)** RNA levels of ASIC1a and ASIC2a were measured by real-time qualitative PCR. RNA levels of ASIC1a and ASIC2a were significantly decreased in adult rats as compared to their neonatal rats. The RNA expression levels of ASIC2a is higher than ASIC1a. RNA level of ASIC1a of neonatal rats was set as 1.0, all others were normalized to the ASIC1a of neonatal rats. *p < 0.05, **p < 0.01, ***p < 0.001, n = 4. **(b)** Protein of ASIC1 and ASIC2a extracted from rat medulla (20 μg) were detected by Western-blotting, β-actin was served as a control. **(c)** Statistical data of (b) and the relative protein levels of ASIC1 and ASIC2a were decreased in adult v.s. neonatal samples. The protein levels of ASICs in neonatal rats were set as control. ***p < 0.001, n = 4.

As shown in Fig. 1a, RNA levels of ASIC1a and ASIC2a were detected in the medulla of neonatal and adult rats by RT-PCR. ASIC2a expression was more abundant than ASIC1a. The ratio of ASIC1a/ASIC2a was approximately 1:7 in neonatal rats and 1:10 in the adult. Additionally, the RNA expression levels of ASIC1a and ASIC2a were lower in adults compared to neonatal rats (n = 4: ASIC1a: 1 ± 0.13 *v.s.* 0.24 ± 0.06, *p* < 0.001; ASIC2a: 7.37 ± 0.84 *v.s.* 2.40 ± 0.67, *p* < 0.001; Fig. 1a). Furthermore, protein levels of ASICs (ASIC1 and ASIC2a) were measured by Western-blotting (Fig. 1b,c). Bands of ASIC1 and ASIC2a both in neonatal and adult rats were shown in Fig. 1b. Consistently, protein levels of ASIC1 and ASIC2a in the VLM were decreased in the adults as compared to neonatal rats (adult compared with neonatal: ASIC1: 1 ± 0.07 *v.s.* 0.70 ± 0.05, *p* < 0.01, n=4; ASIC2a: 1 ± 0.04 *v.s.* 0.74 ± 0.04, *p* < 0.001, n = 4; Fig. 1c). Our data suggests that ASIC1 and ASIC2a are expressed in the medulla of the rat including the VLM and protein levels of both rat ASIC1 and ASIC2a are decreased in the adults as compared to neonatal rats.

### Distribution of ASIC1 and ASIC2a in the medulla of neonatal and adult rats

**Figure 2:**
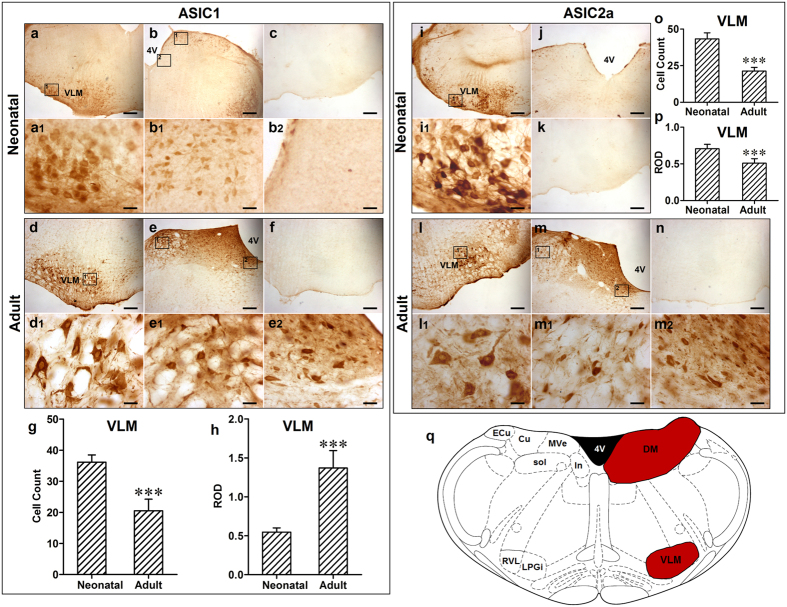
The distribution of ASIC1 and ASIC2a in the medulla of neonatal and adult rats. **(a)** and (**b)** ASIC1 positive cells in the VLM and DM of medulla in neonatal rats, respectively; (**a1**) and (**b1** and **b2**) Representative high power visual field of area in (**a**) and (**b**), respectively. (**c**) Negative control. (**d**) and **(e)** ASIC1 positive cells in the VLM and DM of medulla in adult rats, respectively; (**d1**) and (**e1** and **e2**) Representative high power visual field of area in (**d**) and (**e**), respectively. (**f**) Absorption control, and antibody was pre-absorbed by ASIC1. (**g**) Number of ASIC1 positive cells was decreased in adult rats compared to neonatal rats (***p < 0.001). (**h**) ROD of ASIC1-ir was increased in adult rats compared to neonatal rats (***p < 0.001). (**i**) ASIC2a positive cells in the VLM of medulla in neonatal rats; (**i1**) Representative high power visual field of area in (**i**). (**j**) No ASIC2a positive cell was found in the DM of medulla in neonatal rats. (**k**) Negative control; (**l**) and **(m)** ASIC2a positive cells in the VLM and DM of medulla in adult rats, respectively; (**l1**) and (**m1** and **m2**) Representative high power visual field of area in (**l**) and (**m**), respectively. (**n**) Absorption control, and antibody was pre-absorbed by ASIC2a. (**o**) Number of ASIC2 positive cell in the VLM was decreased in adult rats compared to neonatal rats (***p < 0.001). (**p**) ROD of ASIC2-ir in VLM was also decreased in adult rats compared to neonatal rats (***p < 0.001). (**q**) Coronal diagram of the rat medulla, red area represented distribution of ASIC1 and ASIC2 (right) and the corresponding nuclei are marked out on the left half-side. ***p < 0.001, n = 6. Scale bar = 200 µM (**a**,**b**,**c**,**d**,**e**,**f**,**i**,**j**,**k**,**l**,**m**,**n**), = 25 µM (**a1**, **b1**, **b2**, **d1**, **e1**, **e2**, **i1**, **l1**, **m1**, **m2**). VLM: ventrolateral medulla; DM: dorsal medulla; RVL: rostroventrolateral reticular nucleus; LPGi: lateral paragigantocellular nucleus; In: intercalated nucleus of the medulla; MVe: medial vestibular nucleus; sol: solitary tract; Cu: cuneate nucleus; ECu: external cuneate nucleus.

The localization of ASIC1-immunoreactive (ir) cells was detected in the ventrolateral and dorsal medulla of both neonatal and adult rats (Fig. 2a,b,d and e). According to rat brain atlases (Fig. 2q), ASIC1-ir cells were mainly localized in the VLM including rostroventrolateral reticular nucleus (RVL) and lateral paragigantocellular nucleus (LPGi). Few scattered ASIC1-ir cells (Fig. 2b,e) were found in the dorsal medulla (DM) including the intercalated nucleus of the medulla (In), medial vestibular nucleus (MVe), solitary tract (sol) and external cuneate nucleus (ECu). Additionally, numbers of ASIC1 positive cells were decreased in the VLM of adult rats as compared to neonatal rats (neonatal *v.s.* adult: 36 ± 2 *v.s.* 21 ± 4, *p* < 0.001, n = 6; Fig. 2g) and relative optical density (ROD) of ASIC1-ir cells was increased (neonatal *v.s.* adult: 0.55 ± 0.05 *v.s.* 1.37 ± 0.22, *p* < 0.001, n = 6; Fig. 2h). This suggests that the expression level of ASIC1 per cell was increased, though the total number of cells expressing ASICs was decreased. Distribution of ASIC2a was similar with that of ASIC1 in the medulla of rats except that no ASIC2a-ir cells were observed in the DM of the neonatal rat (Fig. 2i,j,l and m). ROD and ASIC2a positive cells in the VLM were both decreased in adult rats as compared to neonatal rats (neonatal *v.s.* adult: ROD: 0.71 ± 0.06 *v.s.* 0.51 ± 0.06; cell count: 43 ± 4 *v.s.* 21 ± 2, both *p* < 0.001, n = 6; Fig. 2o,p). Additionally, the number of ASIC2a positive neurons in neonatal rats was more than that of ASIC1 (*p* < 0.001, n = 6), yet, they were similar in adult rats. Taken together, our data suggests that ASIC1 and ASIC2a-ir cells are expressed in the rat medulla, including the VLM.

### Co-localization of ASIC1 and ASIC2a in neurons of rat VLM

**Figure 3:**
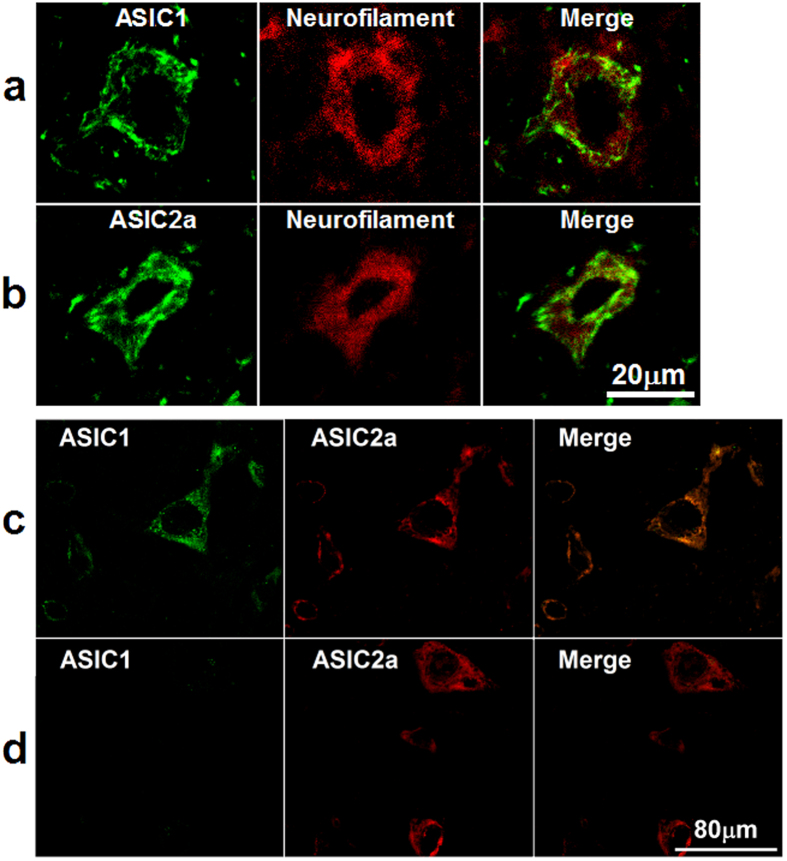
ASIC1 and ASIC 2a were expressed and co-localized in the VLM neurons of adult rat. **(a)** and (**b)** Representative confocal photomicrographs showed co-localization of ASIC1-ir and ASIC2a-ir (green) with neurofilament-ir (red) in the VLM. (**a)** and (**b**) are same scale bar (scale bar = 20 μM). **(c)** Representative confocal photomicrographs showed co-localization of ASIC1 (green) with ASIC2a (red) in the VLM. **(d)** Representative confocal photomicrographs showed there are very few of neurons expressed ASIC2a (red) only. (**c)** and (**d**) are same scale bar (scale bar = 80 μM).

In order to detect whether ASIC1 and ASIC2a are localized in the VLM neurons, ASIC1- or ASIC2a-ir cells were measured in the rat VLM (Fig. 3a,b). ASIC1 and ASIC2a subunits in the rat VLM were co-localized with neurofilaments, the biomarker for neurons (Fig. 3a,b). Further, ASIC1 and ASIC2a were co-localized in the rat VLM (Fig. 3c). Fewer ASIC2a only cells were found in the rat VLM (Fig. 3d).

### ASIC currents in the mouse medulla including the VLM neurons

**Figure 4:**
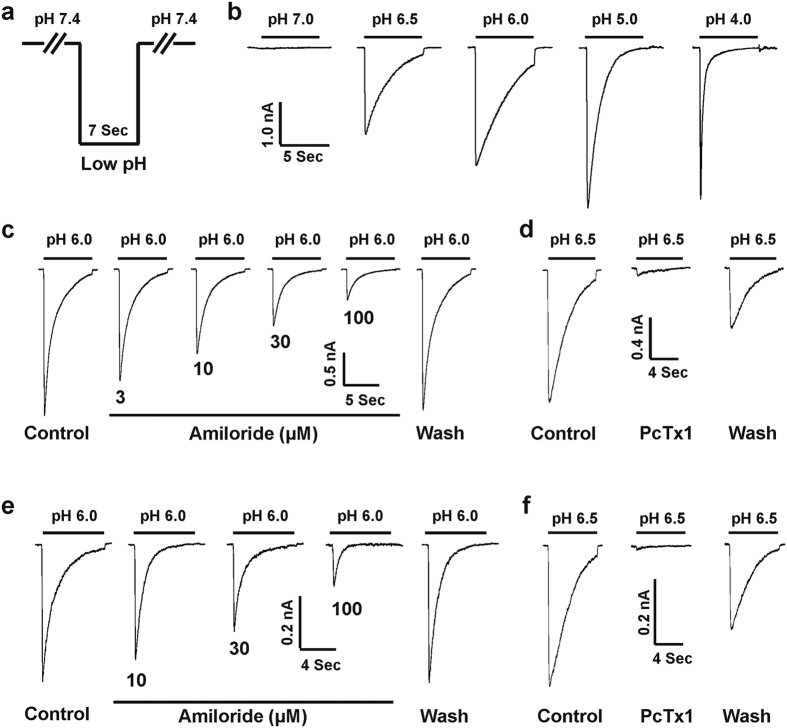
ASIC currents in cultured medulla and acutely dissociated VLM neurons of mouse. (**a**) Fast perfusion for a drop in pH from 7.4 to low value. The perfusion time for low pH value (e.g. 6.0) is 7 s. (**b**) Representative traces showing pH-dependent activation of ASIC currents in cultured medullary neurons and rapid inward currents were recorded with drops in pH from 7.4 to different levels in whole-cell voltage configuration at −60 mV. (**c**) Representative traces showing the dose-dependent inhibition of ASIC currents by amiloride, a non-selective ASIC blocker. The ASIC currents were activated by dropping the pH from 7.4 to 6.0 in cultured medulla neurons. (**d**) Representative traces showing 10 nM PcTx1 significantly inhibited the ASIC currents triggered by dropping the pH from 7.4 to 6.5 in cultured medullary neurons. (**e**) Representative traces showing the dose-dependent inhibition of ASIC currents by amiloride and the ASIC currents were triggered by dropping the pH from 7.4 to 6.0 in acutely dissociated VLM neurons. (**f**) Representative traces showing 10 nM PcTx1 profoundly inhibited the ASIC currents triggered by dropping the pH from 7.4 to 6.5 in acutely dissociated VLM neurons.

ASICs are widely expressed in both the rat and mouse brain^20^,^21^, and amino acid sequences of ASICs in rats and mice are very similar^7^,^20^. Therefore, we further investigated the electrophysiological properties and pharmacological profiles of ASICs in mouse medulla neurons. Both cultured mouse medulla and acutely dissociated VLM neurons were used to characterize the ASICs of mouse medulla including the VLM. Cultured medullary neurons of mice are a suitable surrogate to study ASIC function^17^,^28^. The fast perfusion system was used to switch the barrel from a normal pH of 7.4 to a lower pH (Fig. 4a). As shown in Fig. 4b,a pH-dose response was conducted in cultured mouse medullary neurons with a pH value for half-maximal activation (pH_50_) of 6.60 ± 0.13 (n = 8). Amiloride is widely used to characterize ASICs as a non-selective inhibitor^7^,^39^. We next determined the effects of amiloride on the ASIC current in cultured mouse medullary neurons by decreasing the pH from 7.4 to 6.0. The ASIC current in medullary neurons was dose-dependently and reversibly inhibited by amiloride (Fig. 4c). The IC_50_ value was 21.35 ± 2.17 μM (n = 11) with a Hill coefficient of 1.03 ± 0.06. We also conducted the amiloride blockade on ASIC currents recorded from acutely dissociated mouse VLM neurons. As shown in Fig. 4e, the IC_50_ value of amiloride is 23.70 ± 2.36 μM (n = 8) with the Hill coefficient of 0.99 ± 0.06. There is no significant difference between the IC_50_ value of amiloride on ASIC currents in cultured medulla and acutely dissociated mouse VLM neurons (*p* > 0.05).

Psalmotoxin 1 (PcTx1) selectively inhibits homomeric ASIC1a and heteromeric ASIC1a/2b channels^17^,^40^, therefore we investigated the effects of PcTx1 on ASIC currents in cultured medulla and acutely dissociated VLM neurons in mice, respectively. Application of 10 nM PcTx1 profoundly inhibited the majority of mouse ASIC currents triggered by drops in pH from 7.4 to 6.5 both in cultured mouse medulla (17 out of 28; Fig. 4d) and acutely dissociated mouse VLM (11 out of 18; Fig. 4f) neurons, respectively.

**Figure 5:**
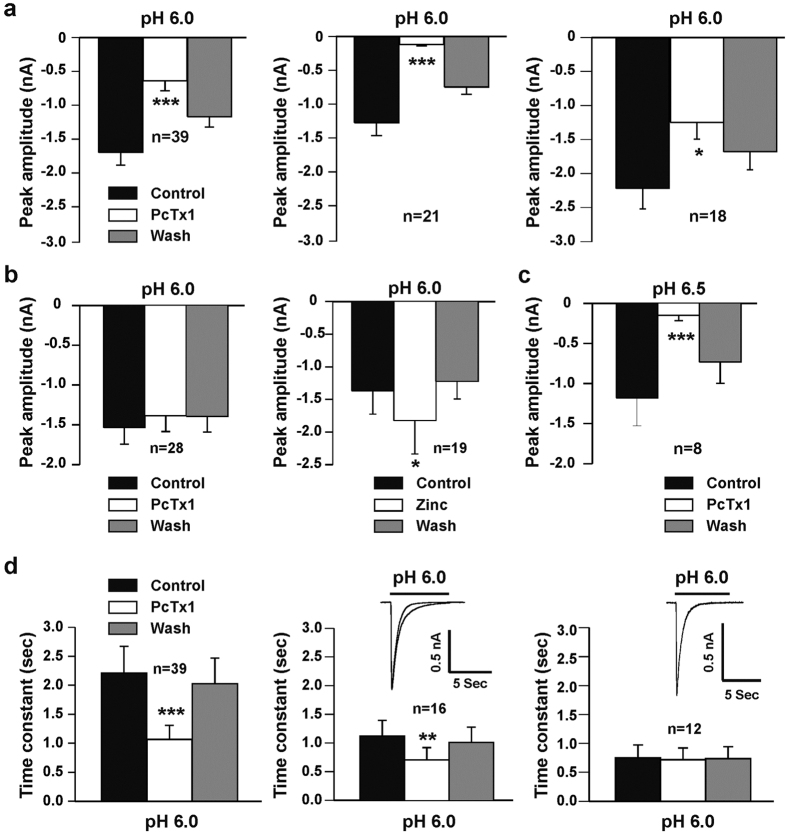
Effects of PcTx1 and zinc on ASIC currents in cultured mouse medullary neurons. (**a**) In one set of neurons tested (n = 39), 10 nM PcTx1 inhibited the mean peak amplitude of ASIC currents (*p* < 0.001; left panel) triggered by dropping pH from 7.4 to 6.0. 10 nM PcTx1 inhibited ASIC currents in 21 out of 39 neurons with over 90% inhibition of the mean peak amplitude (*p* < 0.001; middle panel). In rest of the 18 neurons tested, 10 nM PcTx1 also inhibited ASIC currents with 30% to 90% inhibition of the mean peak amplitude (*p* < 0.05; right panel). (**b**) In another set of 28 neurons tested, 10 nM PcTx1 failed to affect the mean peak amplitude of the ASIC currents activated by drops in pH from 7.4 to 6.0 (*p* > 0.05; left panel). In 19 out of 28 neuron tested, 100 μM zinc potentiated the ASIC currents (*p* < 0.05; right panel). (**c**) Inhibition of the mean peak amplitude of ASIC currents by 10 nM PcTx1 (n = 8; *p* < 0.001). The ASIC currents were triggered by dropping the pH from 7.4 to 6.5 in cultured mouse medullary neurons. (**d**) Effects of PcTx1 on the time constant of ASIC currents. In one set of 39 neurons tested above (**a**), the PcTx1 decreased the time constant (*p* < 0.001; left panel). In another set of 28 neurons tested (**b**), PcTx1 also decreased the time constant in 16 out of 28 neurons (*p* < 0.01; middle panel). The rest of the 12 neurons, PcTx1 didn’t affect the time constant of the ASIC currents (*p* > 0.05; right panel).

We also recorded the ASIC currents triggered by pH drops from 7.4 to 6.0 in a total of 67 cultured mouse medullary neurons according to their sensitivity to PcTx1 and divided them into two groups. One group (39 out of 67) had a peak amplitude of ASIC current that was very sensitive to PcTx1. Bath application of 10 nM PcTx1 significantly inhibited the peak amplitude of ASIC currents triggered by pH drops from 7.4 to 6.0 (n = 39; *p* < 0.001; Fig. 5a, left panel) and 6.5 (n = 8; 8 out of 13; *p* < 0.001; Fig. 5c), respectively. In this set of neurons, we found that one population of the medullary neurons (21 out of 39) is significantly more sensitive to PcTx1 with 90% inhibition of peak amplitude of the ASIC currents activated by pH drops from 7.4 to 6.0 (*p* < 0.001; Fig. 5a, middle panel). In another set of the medullary neurons (18 out of 39), the ASIC currents were also inhibited by 10 nM PcTx1 with 30% to 90% inhibition of the peak amplitude (*p* < 0.05; Fig. 5a, right panel).

In a second group of 28 out of 67 cultured medullary neurons, the peak amplitudes of the ASIC currents weren’t affected by the application of PcTx1 at a concentration of 10 nM (Fig. 5b, left panel; *p* > 0.05). Previous studies have shown that micromolar concentrations of zinc (EC_50_ is close to 110 μM) can potentiate the ASIC2a containing channels^41^,^42^, therefore we tested the contribution of the ASIC2a component in this group. Application of 100 μM zinc potentiated the ASIC currents in 19 out of 28 neurons (*p* < 0.05; Fig. 5b, right panel), indicating that the ASIC2a subunit is present in the majority of this group.

In addition to the peak amplitude, the effect of PcTx1 at a concentration of 10 nM on the time constants 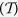 of ASIC currents was also investigated. As illustrated in Fig. 5d, the time constants of ASICs in the first medullary neuron group (39 out of 67) were significantly decreased from 2.21 ± 0.13 s to 1.07 ± 0.07 s (*p* < 0.001; Fig. 5d, left panel). In the second group of medullary neurons (28 out of 67), time constant in response to PcTx1 were different. In 16 out of 28 neurons, the time constants were attenuated from 1.12 ± 0.10 s to 0.70 ± 0.08 s (*p* < 0.01; Fig. 5d, middle panel). The time constants of ASIC currents in remaining neurons (12 out of 28) were unaffected by PcTx1 treatment (0.75 ± 0.08 s to 0.71 ± 0.08 s; *p* > 0.05; Fig. 5d, right panel). Collectively, ASIC currents recorded from mouse medullary neurons reveal different properties in response to PcTx1, a selective inhibitor of homomeric ASIC1a and heteromeric ASIC1a/2b channels^17^,^40^.

### Properties of ASIC currents in acutely dissociated VLM neurons from adult ASIC1 and ASIC2 knock-out (KO) mice

**Figure 6:**
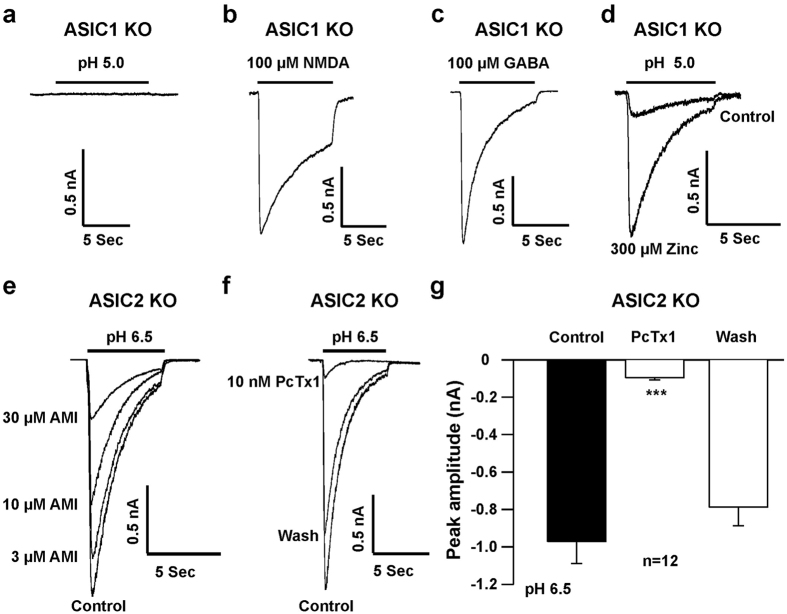
Properties of ASIC current in acutely dissociated VLM neurons from ASIC1 and ASIC2 KO mice. (**a**) Original current trace showing that there was no detectable inward current activated by a drop in pH from 7.4 to 5.0 in the VLM neuron from an ASIC1 KO mouse. In 27 out of 48 recorded VLM neurons there was no response to drop in pH from 7.4 to 5.0. (**b**) Inward current triggered by application of 100 μM NMDA plus 30 μM glycine in the same neuron as (**a**). (**c**) 100 μM GABA triggered inward current in neuron from an ASIC1 KO mouse, which ASIC current cannot be elicited in dropping pH from 7.4 to 5.0. (**d**) Original current trace showing the small inward current activated by a drop in pH from 7.4 to 5.0. The current was potentiated by 300 μM zinc recorded in a neuron from an ASIC1 KO mouse. We observed 21 neurons responding to pH 5.0. (**e**) Representative traces showing the inward current recorded in a neuron from an ASIC2 KO mouse by a drop in pH from 7.4 to 6.5 and amiloride (AMI) dose-dependently inhibited the ASIC current. We recorded 37 neurons in total and all responded to a drop in pH to 6.5. (**f**) Original traces showing that the ASIC current was inhibited by 10 nM PcTx1. (**g**) The peak amplitudes of the ASIC currents were significantly inhibited by 10 nM PcTx1 (n = 12; *p* < 0.001). Acutely dissociated VLM neurons were prepared from two to three month-old ASIC1 or ASIC2 KO mice.

To further characterize the contribution of ASIC subunits in the VLM neurons, we measured the ASIC currents in neurons from adult ASIC1 and ASIC2 KO mice. To verify the properties of ASIC currents observed in cultured medullary neurons, acutely dissociated VLM neurons from 8 to 12 week-old ASIC1 and ASIC2 KO mice were used for whole-cell patch-clamp recordings. The ASIC currents were recorded in a total of 48 neurons from ASIC1 KO mice and there were no detectable currents recorded by pH drops from 7.4 to 6.5 (data not shown), suggesting that the ASIC3 subunit is likely not present in the VLM neurons^21^. As illustrated in Fig. 6a, dropping the pH from 7.4 to 5.0 failed to produce a detectable ASIC current in 27 out of 48 VLM neurons from ASIC1 KO mice, while 100 μM N-methyl-D-aspartate (NMDA) plus 30 μM glycine, which activates glutamate receptors^28^, activated large inward current (Fig. 6b). The inhibitory neurotransmitter GABA at a concentration of 100 μM also induced a large inward current (Fig. 6c). Furthermore, in 21 out of 48 neurons from ASIC1 KO mice, small inward current was elicited by a drop in pH from 7.4 to 5.0, and the current was potentiated by 300 μM zinc (Fig. 6d). This indicates the presence of ASIC2a subunits in 44% (21 out of 48) of the VLM neurons.

Unlike ASIC1 KO mice, ASIC currents were induced in all VLM neurons (n = 37) from ASIC2 KO mice by dropping the pH from 7.4 to 6.5 (Fig. 6e,f). Amiloride dose-dependently inhibited the ASIC currents in these neurons with IC_50_ of 19.80 ± 2.22 μM (n = 6). Bath application of 10 nM PcTx1 significantly inhibited ASIC currents in all neurons tested (n = 12; *p* < 0.001; Fig. 6f,g), indicating that ASIC1a is the major subunit expressed in these neurons.

### Membrane depolarization by activation of ASICs in cultured mouse medulla neurons

**Figure 7:**
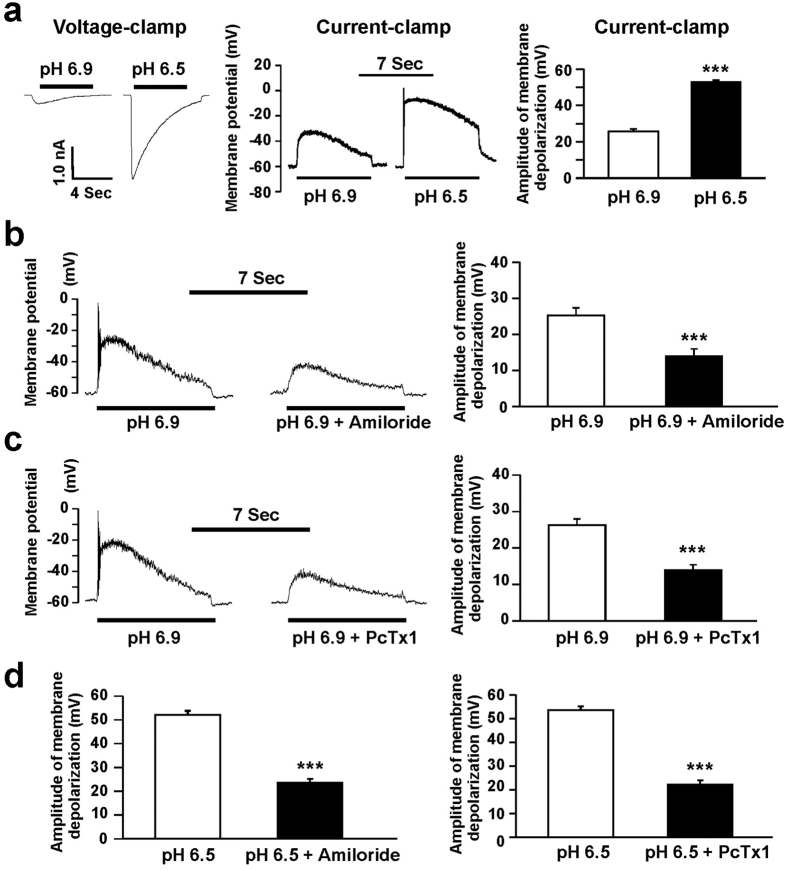
Activation of ASICs induces membrane depolarization in neurons from cultured mouse medullary neurons. **(a)** Under voltage-clamp recording, ASIC currents were activated by a drop in pH from 7.4 to 6.9 or 6.5 in the same neuron (left panel). The ASIC currents were recorded in a total of 12 neurons. The pH 6.9 or 6.5 induced the membrane depolarization was recorded under current-clamp configuration in the same neuron (middle panel). The pH 6.5-induced membrane depolarization was much bigger than pH 6.9 (right panel). **(b)** and (**c)** Representative traces (left panel) and summary data (right panel) showing the membrane depolarization induced by a drop in pH from 7.4 to 6.9 was inhibited by amiloride (100 μM, n = 7, *p* < 0.001) and PcTx1 (10 nM, n = 8, *p* < 0.001), respectively. **(d)** Summary data showing the membrane depolarization induced by a drop in pH from 7.4 to 6.5 was inhibited by amiloride (100 μM, n = 5, *p* < 0.001, left panel) and PcTx1 (10 nM, n = 5, *p* < 0.001, right panel), respectively.

Our next experiment was to define whether a drop in pH elicits membrane depolarization by activating ASICs in cultured mouse medullary neurons. Voltage- and current-clamp recordings were used to record transient inward currents and membrane potentials, respectively^42^. A minor drop in extracellular pH from 7.4 to 6.9, triggered small inward current (Fig. 7a; left panel) under voltage-clamp recording and induced significant membrane depolarization (Fig. 7a, middle panel) from a potential of −60 mV to −34.3 ± 1.5 mV under current-clamp recordings (Fig. 7a; right panel; n = 12, *p* < 0.001). This pH 6.9-mediated membrane depolarization was significantly attenuated by either 100 μM amiloride (from −34.7 ± 2.1 mV to –46.0 ± 2.1 mV; n = 7; *p* < 0.001; Fig. 7b) or 10 nM PcTx1 (from −33.7 ± 1.6 mV to −46.0 ± 1.5 mV; n = 8, *p* < 0.001; Fig. 7c). Further, we dropped pH from 7.4 to 6.5 in cultured medullary neuron, larger inward current was recorded (Fig. 7a; left panel). The pH 6.5-mediated membrane depolarization (Fig. 7a, middle panel) was significantly larger than the pH 6.9-mediated membrane depolarization (Fig. 7a, right panel, n = 12, *p* < 0.001) and this effect was also inhibited by either 100 μM amiloride (Fig. 7d, left panel, n = 5, *p* < 0.001) or 10 nM PcTx1 (Fig. 7d, right panel, n = 5, *p* < 0.001). Taken together, our results demonstrate that ASICs contribute to neuronal excitation of the mouse medulla.

### The antagonists of ASICs attenuated the stimulation effect of acidification on respiration in the rat VLM

**Figure 8:**
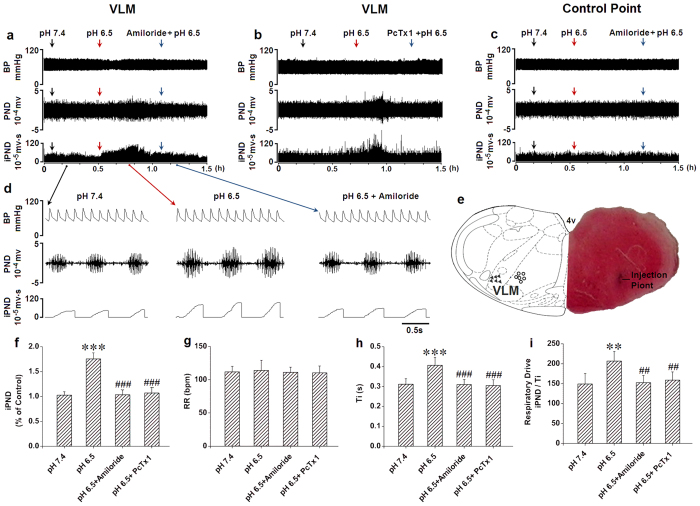
The antagonists of ASICs attenuated the stimulation effect of acidification on respiration in the rat VLM. **(a)** Unilateral microinjection of 0.1 μl ACSF (pH 6.5) into the VLM increased raw PND (middle) and iPND (bottom), but not blood pressure (BP, top). Pre-treatment of amiloride (10 mM, 0.1 μl) blocked the effect. Microinjection of ACSF (pH 7.4) served as the control. **(b)** The records were recorded from the same animal. Pre-treatment of PcTx1 (100 nM, 0.1 μl) blocked the stimulating effect of ACSF (pH 6.5) on PND (middle) and iPND (bottom), but not BP (top). **(c)** Unilateral microinjection of 0.1 μl ACSF (pH 6.5) into the control point had no effect on raw PND, iPND and BP. **(d)** The detailed information from (**a**). **(e)** Histological staining with neutral red: the sky blue spot was the injection site in the VLM, and the injection plot was confirmed by compared with Bregma −12.3 mm coronal diagram in Paxinos & Watson stereotaxic atlas of rat brain. In the diagram, arrowheads represent the injection points and hollow dots signify control points. **(f)** The inhibitory effects of amiloride or PcTx1 on acidification-induced iPND (***p < 0.001 relative to control; ^###^*p* < 0.001 relative to pH 6.5, n = 6). **(g)** Acidification did not alter RR (*p* > 0.05; n = 6). **(h)** Ti was increased by acidification and the effect was inhibited by amiloride and PcTx1 (***p < 0.001 relative to pH 7.4, ^###^*p* < 0.001 relative to pH 6.5, n = 6). **(i)** The respiratory drive (iPND/Ti) was increased by microinjection of pH 6.5 in the VLM and blocked by amiloride and PcTx1 (**p < 0.01 relative to pH 7.4, ^##^*p* < 0.01 relative to pH 6.5, n = 6).

In order to test whether ASICs in the VLM are involved in the central chemoreception, we microinjected an ACSF with different pH levels (7.4, 7.0, 6.5 and 6.0) into rat VLM *in vivo* to observe the effects of phrenic nerve discharge (PND) and integrated PND (iPND) on respiration (Supplementary Fig. S1a). Microinjection of pH 6.5 and pH 6.0, but not pH 7.4 or pH 7.0 triggered significant changes on iPND (Supplementary Fig. S1b), inspiratory time (Ti, Supplementary Fig. S1d) and respiratory drive (integrating value of iPND/Ti, Supplementary Fig. S1e). None of these acidic solutions significantly altered the respiratory rate (RR, Supplementary Fig. S1c). We selected pH 6.5 for further pharmacological studies because it’s profound effects on respiration. The stimulatory effect by pH 6.5 was blocked by microinjection of 10 mM amiloride (Fig. 8a) or 100 nM PcTx1 (Fig. 8b) in the rat VLM. Acidification outside of the VLM had no effect on iPND (Fig. 8c). Microinjection points were verified histologically (Fig. 8e). The stimulation became apparent immediately (within 1 min) following acidification, reaching a peak at 20 to 25 min and returning to baseline within 35 min (Fig. 8a,b). At the peak response, iPND was increased by approximately 75% (from 1.03 ± 0.07 to 1.75 ± 0.12 arbitrary unit, *p* < 0.001, n = 6, Fig. 8f). Though acidification of VLM had no effect on RR (Fig. 8g), Ti was prolonged from 0.31 ± 0.03 to 0.41 ± 0.04 s (*p* < 0.001, n = 6, Fig. 8h). The respiratory drive (integrating value of iPND/Ti) was also increased from 149 ± 26 to 207 ± 24 (*p* < 0.01, n = 6, Fig. 8i). Taken together, these results suggest that activation of ASICs in rat VLM contributes to central chemoreception.

## Discussion

ASICs are proton-gated ion channels and are widely expressed in the brain^7^,^19^,^43^. It was reported that ASICs participate in a variety of physiological and pathological processes including learning/memory, taste sense, hearing, nociception, mechanosensation, and ischemic neuronal injury^4^,^5^,^6^. The central chemoreflex is thought to adjust the activity of the central pattern generator in order to maintain arterial PCO_2_ within a narrow range^1^. There is a common agreement that chemoreceptors located in the VLM in the brain mainly detect change of CO_2_/pH while the peripheral chemoreceptors, chiefly the carotid bodies, are critical for detecting PO_2_ in the blood^44^. Central chemoreceptors monitor the hydrogen ion (H^+^) concentration of cerebrospinal fluid (CSF), including the brain interstitial fluid. CO_2_ readily penetrates membranes, including the blood-brain barrier, whereas H^+^ penetrates slowly. Any increase of H^+^ concentration in CSF stimulates respiration, but how to detect the change in extracellular pH is still unclear. Though recent evidence indicates that additional chemoreceptors are located in the NTS, the locus ceruleus (LC) and hypothalamus^37^,^38^, where different central chemoreceptors are an interdependent system as postulated in the “distributed chemoreception theory”, the VLM is recognized as a specialized region for central chemoreceptors. So we focus on the effects of ASICs on the VLM neurons *in vivo* and *in vitro* in the present study. Although previous works indicated that ASICs both in the lateral hypothalamus and NTS contribute to central regulation of respiration^37^,^38^, the roles of ASICs in the VLM responsible for chemoreception is unknown. The present study demonstrates that functional ASIC1a and ASIC2 are distributed in rat medulla with different expression levels between neonatal and adult neurons including the neurons of VLM (central chemoreception nucleus), and that stimulation of ASICs in the VLM likely contributes to central regulation of breathing.

The expression of ASICs in the brainstem was demonstrated previously^20^,^21^,^43^ where ASIC1 protein was detected in the rat medulla by Western-blotting^20^,^43^. Recently, mRNA of ASIC1a and ASIC2a was detected in NTS of neonatal rodent medulla^38^. Though expression of ASIC1b, ASIC2a and ASIC3 in the trapezoid body and LPGi of adult rats was estimated^45^, the detailed distribution of ASICs in the medulla is still unclear. In the present studies, we examined the expression of ASIC1a and ASIC2a in the medulla by using RT-PCR, Western blotting and immunochemistry techniques combined with patch-clamp recordings. Further, we analyzed the difference in expression and distribution of ASICs in the VLM between neonatal and adult rats. We found that ASICs (1a and 2a) are expressed in the medulla of rats. ASIC2a is more abundant than ASIC1a in both neonatal and adult rats. The protein levels of ASICs (1 and 2a) are decreased in adult rats. The distribution of ASICs (1 and 2a) is similar in adult rats, but differs in neonatal rats. In the adult rat, ASIC1 and ASIC2a subunits are mainly localized in the VLM including RVLM and LPGi with low expression in the DM including In, MVe, sol and ECu. In the neonatal rat, ASIC1 is expressed in the VLM with high expression and DM with low expression. However, ASIC2a is only detected in the VLM, but not DM in the neonatal rats. ECu and MVe play a crucial role in processing proprioceptive and controlling balance, gaze stabilization, and posture^46^,^47^,^48^. The NTS is an important site of integration of afferents such as those from the heart and lungs^49^. It has been reported that ASIC-like currents of neurons in the NTS are involved in the homeostatic control of breathing *in vivo*^38^. The VLM neurons serve as central chemoreceptors mediating cardiovascular and respiratory responses to hypoxia and/or hypercapnia^50^. Our findings laid the anatomical basis to explore the function of ASICs in the brainstem. The developmental change of ASICs in the medulla might contribute to the development of proprioception, vestibular function and central chemoreceptors.

Our patch-clamp recordings indicate that rapid drops in pH from 7.4 to lower pH levels dose-dependently induced transient inward currents in mouse medulla neurons including the VLM. Furthermore, drops in pH induced membrane depolarization and triggered action potentials. The currents and membrane depolarization were blocked by amiloride, a non-selective ASIC inhibitor, and PcTx1, a selective ASIC inhibitor for homomeric ASIC1a and heteromeric ASIC1a/2b channels. Using zinc as a pharmacological tool to discovery the contribution of ASIC2a component^41^, we found that heteromeric ASIC1a/2a channels also contribute to acid-mediated currents in the medulla. Taken together, our data suggest that ASIC1a and ASIC1a/2 channels are likely responsible for acid-activated currents in medullary neurons including the VLM.

We recorded the ASIC currents in the VLM neurons from adult ASIC1 and ASIC2 KO mice. We found that almost all the neurons in wild-type (WT) mice contain the ASIC1a subunit, which was made evident by the pH6.5 triggered ASIC currents found in the VLM neurons from WT, but not ASIC1 KO mice (data not shown). When pH drops to 5.0 in neurons from ASIC1 KO mouse, ASIC currents were found in around 44% of the VLM neurons. Zinc potentiated ASIC currents elicited by pH 5.0 in 21 out of 48 neurons recorded from ASIC1 KO mice, suggesting that ASIC2a subunits are present in the VLM. In addition, ASIC currents triggered by drops in pH from 7.4 to 6.5 were recorded in all neurons from ASIC2 KO mice and inhibited by PcTx1. Collectively, our data from ASIC KO mice reveal that homomeric ASIC1a and heteromeric ASIC1a/2 channels are main components in the VLM neurons.

We found that acidification of rat VLM by microinjection of ACSF at a pH of 6.5 stimulated breathing, amiloride and PcTx1 inhibited this effect, suggesting that homomeric ASIC1a or heteromeric ASIC1a/2 channels may mainly contribute to the central chemoreception. ASICs are widely expressed in both rat and mouse brain^20^,^21^, this has been confirmed by our present studies. The *in vivo* data by microinjection of selective ASIC inhibitor and anatomical results of rat ASIC expression in the rat VLM were consistent with our data of ASIC currents by patch-clamp recording in the mouse VLM neurons *in vitro*.

Co-expression of ASIC1a and ASIC2a was detected in the VLM, which indicates that heteromeric ASICs are likely responsible for central chemoreception. Our patch-clamp recording of ASIC currents from WT and ASIC KO mouse combined with application of PcTx1 and zinc further confirmed this association. Moreover, the relative abundance of individual ASIC subunits influences the acid sensitivity of neurons^36^. If the ratio of ASIC2a/ASIC1a is 1:2, the rate of desensitization of heteromeric currents is increased. Inversely, if the ratio of ASIC2a/ASIC1a is 2:1, the rate of desensitization is decreased^39^. We also found that the number of ASIC1a positive cells was decreased and ROD of ASIC1a was increased in adult rats. A possible interpretation to these findings is that the expression of ASIC1a per neuron was increased in the adult rat, and the reduction of protein level was attributed to a decline in cell numbers. However, compared to neonates, the number of ASIC2a positive cells, ROD, and protein level were all reduced in adult rats. This result implies a decrease in ratio of ASIC2a/ASIC1a in neurons of in adult rat VLM. However, we do not fully understand why these changes occur in adults as compared to neonatal neurons, and further studies are needed to explore the functions of the various ASICs in the VLM. Inversely, the relative increase of ASIC2a compared to ASIC1a throughout development in spinal cord and cortical neurons from mice was addressed in previous studies^52^,^53^. The reason for this controversy was possibly due to the fact that the developmental changes in ASIC1a and ASIC2a might be regional and species-specific.

The change of ASIC1a and 2a expression in the VLM from neonate to adult may reflect the development of respiratory chemoreception in rats. It has been reported that increased PaCO_2_ increases fetal breathing. Central respiratory chemoreception is established early in development as central chemoreceptors appear to be present within days after birth^54^,^55^. It is known that brainstem–spinal cord preparations from newborn rats responded to hypercapnia by increasing respiratory frequency^54^. In the present study, we found that ASIC1a and ASIC2a are widely expressed in the neonatal rat VLM, which suggested that they may participate in central chemoreception in developmental stages even earlier in the fetal period. It is not surprising that newborn and adult mammals have different respiratory responses to hypoxia and hypercapnia^51^,^52^, since breathing serves no gas-exchange function in the fetus and a rapid adaptation must take place immediately after birth to survive in the extra-uterine environment. Previous studies indicated that the response to CO_2_ was quite vigorous immediately after birth but decreased and reached its lowest point by about postnatal day 8, followed by a steady increase until postnatal day 21, when it seemed to reach the response of mature rats^51^. The data showed that the final adult level of CO_2_ sensitivity was less than half that of one day old rats.

More importantly, it was reported that unlike developmental pattern of ventilatory response to hypercapnia *in vivo*, neurons in the LC have a similar response to hypercapnia in rats aged postnatal one day to adult, which indicated that neurons from other chemosensitive regions such as VLM may determine the CO_2_ sensitivity of ventilation^51^. We found that the number of ASIC1a and ASIC2a positive cells as well as protein level of them in the VLM was decreased in adults as compared to neonatal rats. That means that the number of chemosensitive neurons is likewise decreased in adult rats. In addition, the ratio of ASIC2a/ASIC1a is decreased in adult rats, which indicated that the rate of desensitization of ASICs is increased. The changes to the hypercapnia respiratory response may be partly explained by the change in expression of ASIC1a and ASIC2a in the VLM of adult and neonatal rats. Therefore, further experiments are warranted to determine the relationship between the expression of ASIC1a and ASIC2a and respiratory chemoreception in the context of neuronal development.

In summary, we have shown that ASICs including ASIC1 and ASIC2a are expressed in the rat VLM and co-expressed with each other in neurons of the rat VLM. The expression level of ASICs was reduced in adult rats as compared to neonatal rats. Our data from patch-clamp recordings supported the functional ASICs in the mouse medulla and showed that ASIC1a and ASIC1a/2 channels are likely responsible for acid-induced currents in the mouse medulla including the VLM. Furthermore, we demonstrated that ASICs in the VLM contribute to central chemoreception. However, additional studies are needed to further elucidate which ASICs are involved in chemical regulation of breathing during acidosis.

## Methods

### Animals

Neonatal (less than postnatal 7 days) Sprague-Dawley (SD) rats and adult (ranged from 8 to 10 weeks old) male SD rats were provided by the Experimental Animal Center of Chinese Academy of Sciences in Shanghai. ASIC1 and ASIC2 KO mice with a congenic C57BL/6 J background were obtained from Drs. Wemmie and Welsh’s laboratory at the University of Iowa as gifts and subsequently bred in the Laboratory Animal Research Core of the University of Missouri-Kansas City. The C57BL/6 J WT mice were purchased from Jackson Laboratory (Bar Harbor, ME, USA). For the acutely dissociated neurons, 8 to 12 weeks old WT and ASIC KO mice were used. For the medullary cell culture, E16 pregnant WT female mice were used. Animals were maintained on a 12/12 h light/dark cycle with the lights turned on at 7:00 am. The housing environment was maintained at a temperature of 23 °C and humidity of 50% ± 10% with tap water and regular chow available *ad libitum*. All efforts were made to minimize animal suffering and reduce the number of animals used. All animal use procedures were in strict accordance with the US National Institutes of Health for the Care and Use of Laboratory Animals and were approved by the Institutional Animal Care and Use Committee of the University of Missouri-Kansas City and Fudan University Shanghai Medical College.

### Real time PCR

The medulla of adult and neonatal SD rats (n = 4, respectively) were dissected. Total RNAs were extracted with Trizol reagent (ThermoFisher Scientific, USA). First-strand cDNAs were then synthesized from 0.5 μg total RNA in 10 μl volume by reverse transcription using oligo (dT)_12–18_ and Superscript II (TOYOBO, Japan) according to the manufacturer’s protocol. PCR reactions were performed using a 20 μl reaction volume containing 0.25 μl of cDNAs, 10 μl SYBR-green PCR master mixture (TOYOBO, Japan), and 250 nmol/L each primer. The target gene and their primer sequences are shown as follows: ASIC1a (Forward: 5′-GCCTCCGCCAAGTACCTG-3′, Reverse: 5′-CACCCAACAGCCCTGCG ATCT-3′, NM_024154.2); ASIC2a (Forward: 5′-AAGTTGCTGCCTTACTTGGTG-3′, Reverse: 5′-TCTTTGCCAAGCAGGTCTAAT-3′, NM_012892.2), GAPDH (Forward: 5′-AAGGTGGTGAAGCAGGCGGC-3′, Reverse: 5′-GAGCAATGCCAGCCCCAGCA-3′). The PCR amplification consisted of 40 cycles of denaturation (94 °C, 15 seconds), annealing (60 °C, 30 s) and extension (72 °C, 30 s). All the samples were assayed in one assay in our study. The relative quantification of gene expression was analyzed from the measured threshold cycles (Ct). Ct values of ASIC genes were subtracted from the Ct values of GAPDH to yield ΔCt. The average ΔCt of ASIC1a in adult group was subtracted from the ΔCt of all samples to gain ΔΔCt. The fold induction of the mRNA levels of ASICs was calculated using the formula 2^−ΔΔCt^. RNA level of ASIC1a of neonatal rats was set as 1.0, all others were normalized to the ASIC1a of neonatal rats.

### Western blotting

Six adult or neonatal SD male rats were used in the Western blot and the medulla of each was dissected. Tissue samples were placed immediately in liquid N_2_ and later kept at −70 °C until further analysis. Protein samples from rat brain were prepared by homogenization in lysis buffer: 50 mM Tris-HCl pH6.8, 2% sodium dodecyl sulfate (SDS), 1% glycerol, protease inhibitor cocktail (Promega, USA). Homogenates were spun at 12000 g in 4 °C for 15 min and the supernatants were used as a source of protein. Protein concentration was measured with the BCA-100 protein quantitative analysis kit. Equal amounts of protein (20 μg) were resolved in 10% SDS-polyacrylamide gels and transferred to a polyvinylidene fluoride membrane (Millipore Corp., Bedford, MA, USA) for Western blot analysis. After blocking the membranes with 5% nonfat milk in Tris buffer saline Tween 20 (TBST: 20 mM Tris, pH 7.6, 120 mM NaCl, 0.1% Tween), they were incubated with a 1:1000 dilution of the specific purified rabbit anti rat ASIC1 antibody (Alomone Lab, Israel), rabbit anti rat ASIC2a antibody (Alomone Lab, Israel) or mouse anti rat anti-β-actin antibody (Sigma-Aldrich, USA) at 4 °C for 12 h, followed by extensive washes with TBST. A 1:5000 dilution of horseradish peroxidase-conjugated anti-rabbit IgG or anti-mouse IgG (Beyotime Institute of Biotechnology, China) was added and membranes were incubated for 1 h at room temperature. Chemiluminescent signals were generated by the addition of enhanced Chemiluminescent Substrate (Santa Cruz biotechnology Inc, USA) and detected on a radiographic film. β-actin was measured at the band of 35 kd. The ASIC1 and ASIC2a were detected at the band of 70 kd and 65 kd, respectively.

### Immunohistochemistry

Adult (6~8 w) and neonatal (1–3 d) SD rats (n = 6) were anesthetized with urethane (1 g. kg^−1^) by intraperitoneal injection and perfused through the left ventricle with normal saline followed by 4% paraformaldehyde in 0.1 M phosphate buffer, pH 7.4. After perfusion, brains were removed and post-fixed by immersion in the same fixative overnight. The medullary tissues were dissected, equilibrated in graded sucrose solution (20% to 30%) and coronal sectioned at 30 μm in one to five serial orders on Leica freezing microtome.

For immunohistochemistry ABC method, slides were washed in 0.01 M PBS, pH 7.4. The slides were incubated with first antibody of ASIC1 or ASIC2a (Alomone Lab, Israel, rabbit anti rat IgG, 1:100) diluted in 1% bovine serum albumin buffer (1% BSA) and the controls were in 1% BSA or in the antibody which was absorbed by relevant peptides before use for 24 h in 4 °C. All dilutions had been established by preliminary titration. After 3 washes in 0.01 M PBS, The slides were incubated in 1% BSA for 1 h to block background staining, and the reaction was detected with avidin-biotin-HRP complex (ABC) immuno detects kit (Sino-American Biotechnology Co., USA). Sections were transferred on glasses, dried in the open-air and mounted after dehydration and transparence, then examined with light microscopy. Number and ROD [(positive staining-background)/background] of positive stained cells were calculated in six representative sections from each animal in a blinded manner to the treatment by the software of Image Measure Version 1.0 (Department of Physiology & Pathophysiology, Shanghai Medical College, Fudan University, Shanghai, China).

For double immunofluorescence technique, slides were washed in 0.05 M Tris–Saline Buffer, pH 7.6 containing 0.1% Triton X-100 (TBSTx). The slides were incubated with a mixture first antibody of ASIC1/2a with neurofilament (Chemicon, USA), rabbit anti rat IgG, ASIC1 and ASIC2a diluted in 1% BSA for 24 h. All dilutions had been established by preliminary titration. After 3 washes in TBSTx, The slides were incubated in 1% mixture serum of donkey and goat for 1 h to block background staining, and then the reactions were detected with a mixture of donkey anti rabbit IgG conjugated with cy3 (Chemicon, USA) and donkey anti goat IgG conjugated with FITC (Santa Cruz Biotechnology, USA) diluted 1:200 in 1% BSA for 1 h in the dark. After 3 washes in TBSTx, the slides were air-dried, and mounted in antifading medium (Beyotime Institute of Biotechnology, China), and examined with confocal laser scanning microscope (Zeiss 510, USA).

### Primary medullary neuronal cultures

Primary cultures of mouse medullary neurons were prepared according to previously described techniques^42^. Briefly, time-pregnant (embryonic day 16) C57BL/6 J mice were anaesthetized with halothane followed by cervical dislocation. Fetuses were rapidly removed and placed in Ca^2+^- and Mg^2+^-free cold Hank’s solution. The medulla was dissected under the microscope and incubated with 0.05% trypsin-EDTA for 10 min at 37 °C, followed by trituration with fire-polished glass pipettes, and plating on poly-L-ornithine-coated 35 × 35 mm culture dishes at a density of 1 × 10^6^ cells per dish. Medullary neurons were cultured with neurobasal medium supplemented with B27 and maintained at 37 °C in a humidified 5% CO_2_ atmosphere incubator. Cultures were fed with neurobasal medium plus B27 twice weekly and used for electrophysiological recording 10–14 days after plating.

### Acute isolation of mouse VLM neurons from WT, ASIC1 and ASIC2 KO mice

Mouse VLM neurons were acutely isolated according to our previously described technique^42^. Briefly, WT, ASIC1 and 2 KO male mice (C57BL/6 genomic background), 8 to 12 weeks of age, were anesthetized with halothane and sacrificed by decapitation using a guillotine. The whole brain was removed and placed in cold extracellular solution (ECF), and the medulla was subsequently sectioned at 250~300 μm with a microtome (Leica VT 1000 S, Germany). The slices were then incubated in ECF containing 3–5 mg/ml papain (from papaya latex, Sigma-Aldrich Chemical Co., USA) at room temperature for 20 to 30 min. All ECFs were bubbled with 100% O_2_. Following the enzymatic digestion, slices were washed three times and incubated in enzyme-free ECF for at least 30 min before dissociation. To isolate VLM neurons, individual slices were transferred into a 35 mm culture dishes containing 2 ml of ECF and each dish containing each slice was placed on the stage of an inverted phase-contrast microscope for identifying the VLM region. The VLM region of the slice was excised and single cells were mechanically dissociated using two fire polished glass pipettes or fine forceps. Electrophysiological recording of isolated VLM neurons began approximately 30 min after mechanical dissociation.

### Whole-cell patch-clamp recordings

Whole-cell patch-clamp recordings were performed as described previously^42^. Patch electrodes, whose resistances ranged from 4 and 8 MΩ when filled with intracellular solution, were constructed from thin-walled borosilicated glass (1.5 mm diameter, WPI, Sarasota, FL, USA) on a two-stage puller (PC-10, Narishige, Tokyo, Japan). Whole-cell currents or membrane potentials were recorded using Axopatch 200B amplifier (Molecular Devices, CA, USA). Data were filtered at 2 kHz and digitized at 5 Hz using Digidata 1440 DAC units (Molecular Devices, CA, USA). The on-line acquisition was done using pCLAMP 10 software (Molecular Devices, CA, USA).

In order to avoid current desensitization, ASIC channels were triggered by reducing the pH from 7.4 to specific target levels (e.g. 6.0) every 2 min. The duration of pH drops in low pH values (e.g. pH 6.0) is 7 s (see Fig. 4a). During each experiment, a voltage step of −10 mV from the holding potential was applied periodically to monitor the cell capacitance and the access resistance. Recordings in which either the access resistance or the capacitances changed by more than 10% during the experiment were excluded from data analysis.

### Solutions and compounds for patch-clamp recording

Standard ECF contained (mM): 140 NaCl, 5.4 KCl, 2.0 CaCl_2_, 1.0 MgCl_2_, 20 Hepes, 10 Glucose (pH 7.4; 320–330 mOsm). For solutions with pH of 6.0 or lower, MES was used instead of HEPES for more reliable pH buffering^42^. For voltage-clamp recordings, the pipette solution contained (mM): 140 CsF, 10 Hepes, 11 EGTA, 2 TEA, 1 CaCl_2_, 2 MgCl_2_ and 4 K_2_ATP (pH 7.2~7.3; 290~300 mOsm). For recording of GABA currents, CsF in the pipette was replaced with CsCl. For current-clamp recording, CsF in the pipette solution was replaced with K-gluconate. Psalmotoxin 1 (PcTx1) was purchased from Peptides International Inc (Louisville, KY); other chemicals were purchased from Sigma-Aldrich (Sigma-Aldrich Chemical Co., St. Louis, MO, USA). A multi-barrel perfusion system (SF-77, Warner Instrument Co., CT, USA) was employed to achieve a rapid exchange of ECF.

### Phrenic Nerve Discharge Recording

The method has been used in our previous publication^37^. Activities in the left phrenic nerve of rats were recorded with platinum bipolar electrodes, pre-amplified and bandpass filtered (5 KHz) by a Polygraph System (Nihon Kohden, Japan), digitized by Bioelectric signals processing system (SMUP-E, Shanghai Medical College, Fudan University, Shanghai, China). The experiments were started after stabilization of the phrenic activity (about 30 min) and then the PND was recorded and analyzed using MFLab 200 software (Shanghai Medical College, Fudan University, Shanghai, China). The iPND was obtained by a moving average of the phrenic signal. Each iPND over the period of Ti was used to assess the respiratory drive. Respiration rate was computed from the PND.

### Microinjections

Unilateral microinjection into VLM of rats (12.3 mm posterior, 2.2 mm lateral, and 10 mm dorsal from bregma), at a volume of 0.1 μl, was carried out stereotacically and sequentially with a 27-gauge stainless steel (internal cannula connected to a 1 μl microliter syringe). An injection point outside of VLM served as control (12.3 mm posterior, 1.4 mm lateral, and 9.8 mm dorsal from bregma). The injection position was verified by histology at the end of the experiment. Test agent, a nonselective ASICs inhibitor (10 mM amiloride) and selective ASIC1a inhibitor (100 nM PcTx1) were freshly prepared in ACSF immediately before administration. ACSFs were prepared at different pHs (7.4, 7.0, 6.5 and 6.0). Co-microinjection was performed with amiloride first followed by the effective pH immediately. ACSF with pH 7.4 served as the vehicle and volume control with the composition (mM): NaCl 130, NaHCO_3_ 26, KCl 5, CaCl_2_ 2.6, MgSO_4_ 1.2, NaH_2_PO_4_ 1.6, glucose 11 and sucrose 10. At the end of the experiment, 0.1 μl pontamine sky blue was injected into the injection point. Brains were removed, fixed, frozen, coronally sectioned (30 μm), and stained with neutral red for histological verification.

### Data analysis

Statistical analysis was done with Stata or SigmaPlot software. Data are reported as means ± standard error of the mean (Mean ± SEM). For Western blotting, the protein level of ASICs in each sample was calculated as ASICs/β-actin. The protein level of ASIC1 and ASIC2 in the neonatal rats served as control. The average level of ASIC1 and ASIC2 in neonatal rats were normalized as 1. The ratio of adult over neonatal rats represents the relative protein level of ASICs in adult rats. For phrenic nerve discharge recording, iPND at pH7.4 was normalized as 1. The activation of phrenic nerve was computed as the ratio of iPND at other pHs over iPND at pH7.4. *t* test and one-way parametric ANOVA followed by all pair-wise multiple comparisons (Bonferroni method) were used as appropriate. Significance were set at *p < 0.05, **P < 0.01 or ^##^*P* < 0.01, ***p < 0.001 or ^###^*P* < 0.001.

For pH activation curves, the ECF flowing out of one barrel of the perfusion system was pH 7.4 while the ECF flowing out of the second barrel was switched to pH 7.0, 6.5, 6.0, 5.0 and 4.0 sequentially using the SF-77B fast perfusion system (Warner Instrument Co., CT, USA). Acid-triggered currents at each pH were normalized to the peak current activated at a pH of 5.0. Normalized values were fitted to the Hill equation using SigmaPlot 10 software to obtain pH_50_ values and Hill coefficients.

To determine the time constant of the fit of the desensitizing portion of the ASIC currents triggered by a drop in pH from 7.4 to 6.0, pH 6.0-activated currents with or without PcTx1 treatment were fitted by a single, standard exponential equation using Clampfit 10.2.

## Additional Information

**How to cite this article**: Song, N. *et al*. Acid-sensing ion channels are expressed in the ventrolateral medulla and contribute to central chemoreception. *Sci. Rep.* **6**, 38777; doi: 10.1038/srep38777 (2016).

**Publisher's note:** Springer Nature remains neutral with regard to jurisdictional claims in published maps and institutional affiliations.

## Supplementary Material

Supplementary Information
