# Supplementary material for: Acid-sensing ion channels are expressed in the ventrolateral medulla and contribute to central chemoreception
Source: Sci Rep. 2016 Dec 9;6:38777. doi: 10.1038/srep38777 (PMC5146928; doi:10.1038/srep38777)

Acid-sensing ion channels are expressed in the ventrolateral medulla and contribute to central chemoreception

Nana Song[1, 2, *], Ruijuan Guan[1, *], Qian Jiang[3, *], Comron J. Hassanzadeh[3], Yuyang Chu[3], Xiaomei Zhao[1], Xia Wang[1], Dawei Yang[1, 4], Qijun Du[1], Xiang-Ping Chu[3, 5], & Linlin Shen[1, 6]

Supplementary Fig. S1. Effect of different levels of acidification on respiration in the rat VLM *in vivo*. (a) Unilateral microinjected 0.1 μl ACSF with different pH value (pH 7.4, 7.0, 6.5 and 6.0) was into the VLM. ACSF with pH 7.0, 6.5 and 6.0 increased raw PND (top) and iPND (bottom), compared with ACSF in pH 7.4. **(b-e)** Statistical data showing that effects of different acidification on iPND (b), RR (c), Ti (d), respiratory drive (iPND/Ti, e). The stimulating effect of ACSF with pH 7.0 was not significant as compared to pH 7.4 ($P > 0.05$) and that of pH 6.5 and 6.0 was significant. * $p < 0.05$, ** $p < 0.01$, *** $p < 0.001$ relative to pH 7.4, n = 6.

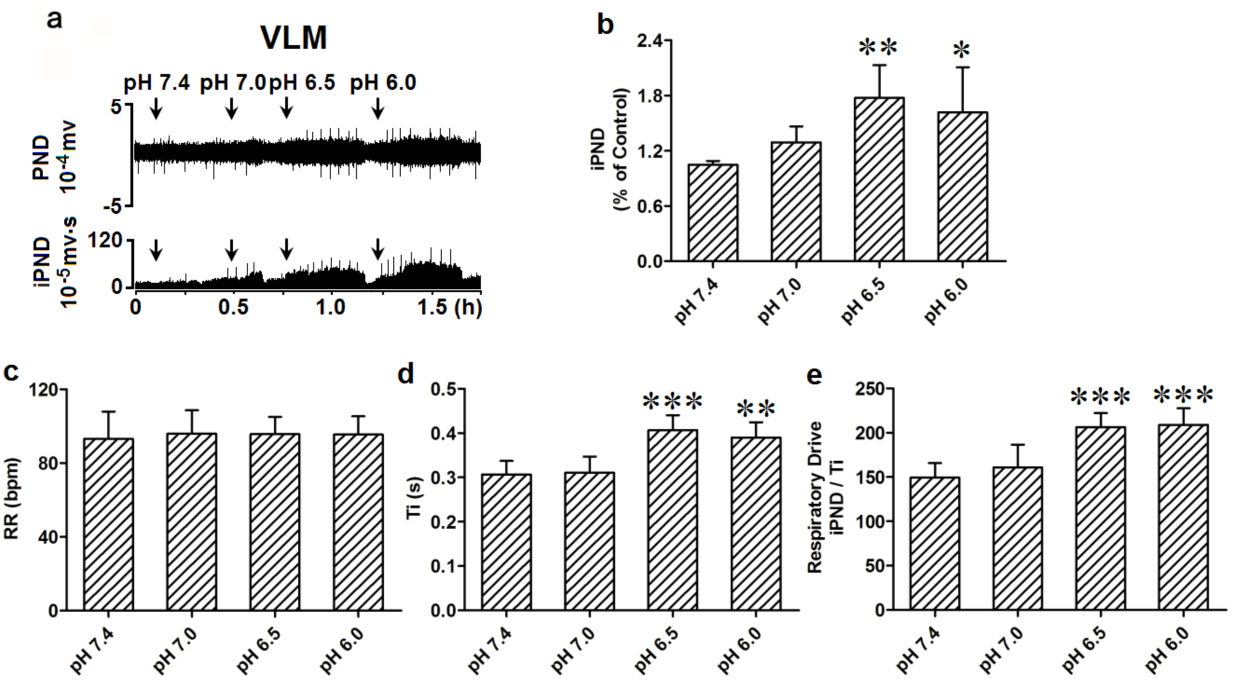

Supplement: Supplementary Information [file srep38777-s1.pdf]
